# 2-[(*E*)-2-(3,4-Di­chloro­benzyl­idene)hydrazin-1-yl]quinoxaline

**DOI:** 10.1107/S1600536814000415

**Published:** 2014-01-11

**Authors:** Thais C. M. Noguiera, Alexandra C. Pinheiro, Marcus V. N. de Souza, James L. Wardell, Edward R. T. Tiekink

**Affiliations:** a Fundação Oswaldo Cruz, Instituto de Tecnologia em, Fármacos–Farmanguinhos, R. Sizenando Nabuco, 100, Manguinhos 21041-250, Rio de Janeiro, RJ, Brazil; b Chemistry Department, University of Aberdeen, Old Aberdeen AB24 3UE, Scotland; c Department of Chemistry, University of Malaya, 50603 Kuala Lumpur, Malaysia

## Abstract

The 21 non-H atoms of the title compound, C_15_H_10_Cl_2_N_4_, are almost planar (r.m.s. deviation = 0.032 Å); the conformation about the N=C bond [1.277 (6) Å] is *E*. In the crystal, zigzag supra­molecular chains along the *c* axis (glide symmetry) are formed *via* N—H⋯N hydrogen bonds. These associate along the *b* axis by π–π inter­actions between the fused and terminal benzene rings [inter­centroid distance = 3.602 (3) Å] so that layers form in the *bc* plane.

## Related literature   

For the use of quinoxaline compounds as dyestuffs and biological agents, see: Mielcke *et al.* (2012 ▶); Mamedov & Zhukova (2012 ▶); Rodrigues *et al.* (2014 ▶). For a related hydrazone structure, see: de Souza *et al.* (2013 ▶).
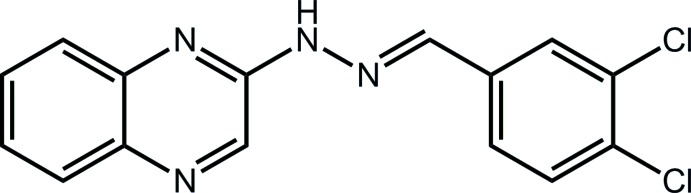


## Experimental   

### 

#### Crystal data  

C_15_H_10_Cl_2_N_4_
*M* *_r_* = 317.17
Monoclinic, 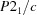

*a* = 16.0284 (11) Å
*b* = 6.9756 (4) Å
*c* = 12.4127 (9) Å
β = 96.043 (7)°
*V* = 1380.12 (16) Å^3^
*Z* = 4
Mo *K*α radiation
μ = 0.47 mm^−1^
*T* = 120 K
0.20 × 0.13 × 0.03 mm

#### Data collection  

Rigaku RAXIS conversion diffractometer
Absorption correction: multi-scan (*CrystalClear-SM Expert*; Rigaku, 2012 ▶) *T*_min_ = 0.654, *T*_max_ = 1.000
7037 measured reflections
2385 independent reflections
1670 reflections with *I* > 2σ(*I*)
*R*_int_ = 0.043

#### Refinement  

*R*[*F* ^2^ > 2σ(*F* ^2^)] = 0.060
*wR*(*F* ^2^) = 0.219
*S* = 1.20
2385 reflections
193 parameters
H atoms treated by a mixture of independent and constrained refinement
Δρ_max_ = 0.76 e Å^−3^
Δρ_min_ = −0.65 e Å^−3^

### 

Data collection: *CrystalClear-SM Expert* (Rigaku, 2012 ▶); cell refinement: *CrystalClear-SM Expert*; data reduction: *CrystalClear-SM Expert*; program(s) used to solve structure: *SHELXS97* (Sheldrick, 2008 ▶); program(s) used to refine structure: *SHELXL97* (Sheldrick, 2008 ▶); molecular graphics: *ORTEP-3 for Windows* (Farrugia, 2012 ▶) and *DIAMOND* (Brandenburg, 2006 ▶); software used to prepare material for publication: *publCIF* (Westrip, 2010 ▶).

## Supplementary Material

Crystal structure: contains datablock(s) general, I. DOI: 10.1107/S1600536814000415/hg5372sup1.cif

Structure factors: contains datablock(s) I. DOI: 10.1107/S1600536814000415/hg5372Isup2.hkl

Supporting information file. DOI: 10.1107/S1600536814000415/hg5372Isup3.cml

CCDC reference:

Additional supporting information:  crystallographic information; 3D view; checkCIF report

## Figures and Tables

**Table 1:** Hydrogen-bond geometry (Å, °)

*D*—H⋯*A*	*D*—H	H⋯*A*	*D*⋯*A*	*D*—H⋯*A*
N2—H2*N*⋯N4^i^	0.92 (5)	2.10 (5)	3.013 (5)	171 (4)

Symmetry code: (i) 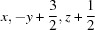.

**Fig. 1.**
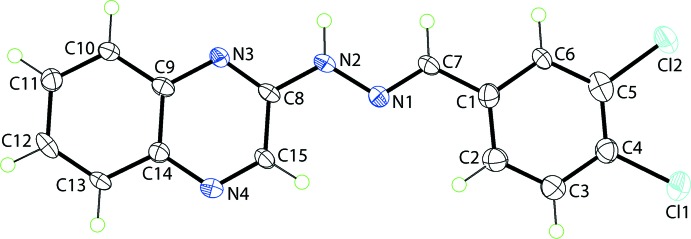
The molecular structure of (I) showing the atom-labelling scheme and displacement ellipsoids at the 50% probability level.

**Fig. 2.**
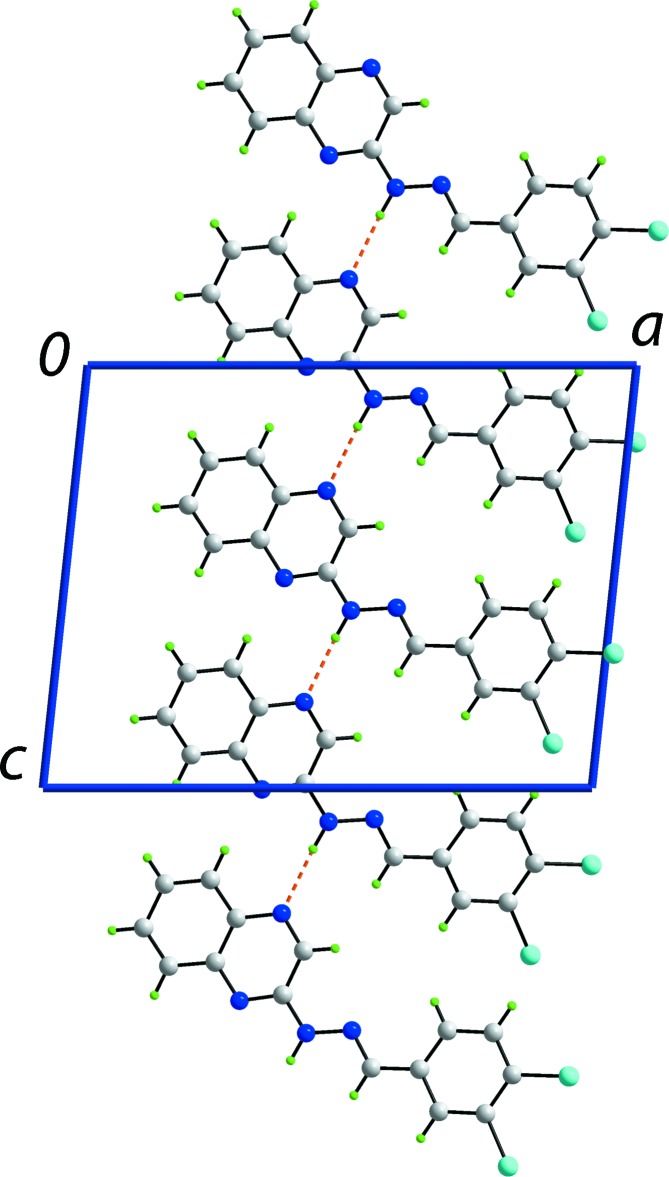
A view of the supramolecular zigzag chain along the c axis in (I). The N—H···H hydrogen bonds are shown as orange dashed lines.

**Fig. 3.**
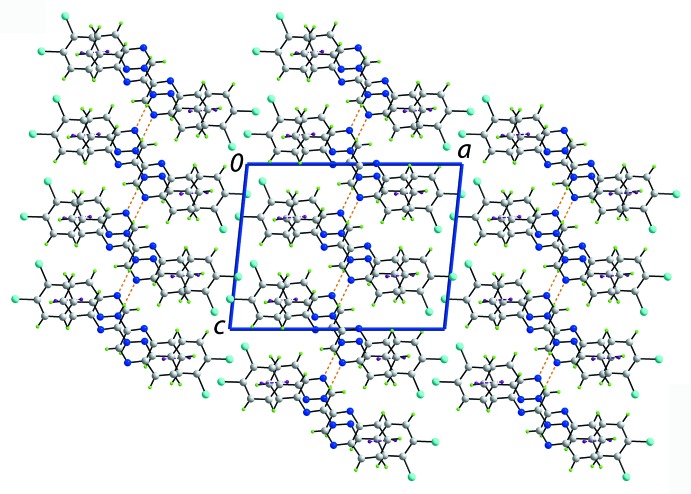
A view in projection down the b axis of the unit-cell contents for (I). The N—H···H and π—π interactions are shown as orange and purple dashed lines, respectively.

## supplementary crystallographic information

### 2.1. Synthesis and crystallization

A solution of 2-hydrazinylquinozaline (1 mmol) and 3,4-di­chloro­benzaldehyde (1.05 mmol) in EtOH (10 ml) was stirred at room temperature for 24 h and rotary evaporated. The residue was washed with ice-cold EtOH (3 ×) and recrystallized from its MeOCH_2_CH_2_OH solution. Yield: 95%. M.Pt: 528–529 K. ^1^H NMR (400 MHz, DMSO-d_6_): δ 11.86 (1H, s, NH); 9.15 (1H, s, H3); 8.11 (1H, s, CH); 8.04 (1H, d, *J* = 2.0; H2'); 7.93 (1H, d, *J* = 8.2, H5); 7.77 (1H, dd, *J* = 8.4 and *J* = 2.0, H6'); 7.68 (3H, m, H5', H7 and H8); 7.51 (1H, dt, *J* = 8.2 and *J* = 2.0, H6) ppm. ^13^C NMR (100 MHz, DMSO-d_6_): δ 150.0; 140.7; 139.0; 138.0; 136.4; 135.5; 131.6; 131.1; 130.8; 130.3; 128.7; 127.8; 126.3; 126.2; 125.3 ppm. MS/ESI (M—H): 314.9. IR (cm^-1^, KBr): 3437 ν(N—H); 1585 ν(C═N).

### 2.2. Refinement

Intensity data was collected at the National Crystallographic Service, England (Coles & Gale, 2012). The C–bound H atoms were geometrically placed (C—H = 0.95 Å) and refined as riding with *U*_iso_(H) = 1.2*U*_eq_(C). The N-bound H atom was located from a difference map and refined with *U*_iso_(H) = 1.2*U*_eq_(N).

### 3. Results and discussion

Quinoxaline compounds have found uses, mainly as biologically active compounds, but also as dyestuffs (Mielcke *et al.*, 2012; Mamedov & Zhukova, 2012). The biological activities of quinoxaline compounds include anti-bacterial, anti-tubercular, anti-microbial, anti-fungal, anti-malarial, anti-inflammatory, anti-leishmanial, anti-tumour, herbicidal and insecticidal (Mielcke *et al.*, 2012; Mamedov & Zhukova, 2012). In a recent study, an evaluation of the anti-cancer activities of a series of (*E*)-2-(2-benzyl­idene)hydrazinyl)quinoxaline derivatives was reported (Rodrigues *et al.*, 2014). As part of our continuing studies on the structures of biologically active hydrazones (de Souza *et al.*, 2013), we now report the crystal structure of the title compound, (*E*)-2-(2-(3,4-di­chloro­benzyl­idene)hydrazinyl)-quinoxaline, (I).

In (I), Fig. 1, the 21 non-hydorgen atoms comprising the molecule are co-planar with the r.m.s. deviation = 0.032 Å; the maximum deviations from the least-squares plane are 0.066 (5) Å for atom C5 and -0.057 (1) Å for atom Cl2. The conformation about the N1═C7 bond [1.277 (6) Å] is *E*.

In the crystal packing, zigzag supra­moelcular chains (glide symmetry) are formed *via* N—H···N hydrogen bonds, Fig. 2 and Table 1. These chains along the *c* direction are consolidated into supra­molecular layers in the *bc* plane by π—π inter­actions between the (C1–C6) and (C9–C14)^i^ rings [inter-centroid distance = 3.602 (3) Å, inter-planar angle = 2.4 (2)° for symmetry operation *i*: 1-*x*, 1-*y*, -*z*] formed along the *b* direction, Fig. 3.

### Crystal data

**Table:**

C_15_H_10_Cl_2_N_4_	*F*(000) = 648
*M**_r_* = 317.17	*D*_x_ = 1.526 Mg m^−^^3^
Monoclinic, *P*2_1_/*c*	Mo *K*α radiation, λ = 0.71073 Å
Hall symbol: -P 2ybc	Cell parameters from 5450 reflections
*a* = 16.0284 (11) Å	θ = 3.2–29.1°
*b* = 6.9756 (4) Å	µ = 0.47 mm^−^^1^
*c* = 12.4127 (9) Å	*T* = 120 K
β = 96.043 (7)°	Prism, yellow
*V* = 1380.12 (16) Å^3^	0.20 × 0.13 × 0.03 mm
*Z* = 4	

### Data collection

**Table:**

Rigaku RAXIS conversion diffractometer	2385 independent reflections
Radiation source: Sealed Tube	1670 reflections with *I* > 2σ(*I*)
Graphite monochromator	*R*_int_ = 0.043
Detector resolution: 10.0000 pixels mm^-1^	θ_max_ = 25.0°, θ_min_ = 3.2°
profile data from ω–scans	*h* = −19→17
Absorption correction: multi-scan (*CrystalClear-SM Expert*; Rigaku, 2012)	*k* = −8→7
*T*_min_ = 0.654, *T*_max_ = 1.000	*l* = −14→14
7037 measured reflections	

### Refinement

**Table:**

Refinement on *F*^2^	Primary atom site location: structure-invariant direct methods
Least-squares matrix: full	Secondary atom site location: difference Fourier map
*R*[*F*^2^ > 2σ(*F*^2^)] = 0.060	Hydrogen site location: inferred from neighbouring sites
*wR*(*F*^2^) = 0.219	H atoms treated by a mixture of independent and constrained refinement
*S* = 1.20	*w* = 1/[σ^2^(*F*_o_^2^) + (0.1005*P*)^2^ + 2.9896*P*] where *P* = (*F*_o_^2^ + 2*F*_c_^2^)/3
2385 reflections	(Δ/σ)_max_ < 0.001
193 parameters	Δρ_max_ = 0.76 e Å^−^^3^
0 restraints	Δρ_min_ = −0.65 e Å^−^^3^

### Special details

**Table:**

Geometry. All s.u.'s (except the s.u. in the dihedral angle between two l.s. planes) are estimated using the full covariance matrix. The cell s.u.'s are taken into account individually in the estimation of s.u.'s in distances, angles and torsion angles; correlations between s.u.'s in cell parameters are only used when they are defined by crystal symmetry. An approximate (isotropic) treatment of cell s.u.'s is used for estimating s.u.'s involving l.s. planes.
Refinement. Refinement of *F*^2^ against ALL reflections. The weighted *R*-factor *wR* and goodness of fit *S* are based on *F*^2^, conventional *R*-factors *R* are based on *F*, with *F* set to zero for negative *F*^2^. The threshold expression of *F*^2^ > 2σ(*F*^2^) is used only for calculating *R*-factors(gt) *etc*. and is not relevant to the choice of reflections for refinement. *R*-factors based on *F*^2^ are statistically about twice as large as those based on *F*, and *R*- factors based on ALL data will be even larger.

### Fractional atomic coordinates and isotropic or equivalent isotropic displacement parameters (Å^2^)

**Table:**

	*x*	*y*	*z*	*U*_iso_*/*U*_eq_	
Cl1	0.92037 (8)	0.4044 (2)	0.39515 (10)	0.0391 (4)	
Cl2	1.01377 (8)	0.3943 (2)	0.18221 (11)	0.0391 (4)	
N1	0.6096 (2)	0.6684 (6)	0.0737 (3)	0.0234 (9)	
N2	0.5269 (2)	0.7147 (6)	0.0801 (3)	0.0249 (9)	
H2N	0.504 (3)	0.718 (7)	0.145 (4)	0.030*	
N3	0.3991 (2)	0.8161 (5)	0.0034 (3)	0.0206 (9)	
N4	0.4593 (2)	0.8165 (6)	−0.2031 (3)	0.0247 (9)	
C1	0.7406 (3)	0.5710 (7)	0.1655 (4)	0.0256 (11)	
C2	0.7839 (3)	0.5206 (7)	0.2653 (4)	0.0244 (11)	
H2	0.7560	0.5212	0.3291	0.029*	
C3	0.8691 (3)	0.4690 (7)	0.2706 (4)	0.0307 (12)	
C4	0.9107 (3)	0.4672 (7)	0.1782 (4)	0.0281 (11)	
C5	0.8680 (3)	0.5232 (7)	0.0788 (4)	0.0301 (12)	
H5	0.8963	0.5262	0.0154	0.036*	
C6	0.7837 (3)	0.5744 (7)	0.0738 (4)	0.0291 (11)	
H6	0.7549	0.6125	0.0064	0.035*	
C7	0.6523 (3)	0.6206 (7)	0.1623 (4)	0.0235 (11)	
H7	0.6259	0.6175	0.2273	0.028*	
C8	0.4770 (3)	0.7668 (6)	−0.0107 (3)	0.0203 (10)	
C9	0.3477 (3)	0.8652 (6)	−0.0881 (3)	0.0215 (10)	
C10	0.2636 (3)	0.9113 (7)	−0.0786 (4)	0.0225 (10)	
H10	0.2431	0.9085	−0.0095	0.027*	
C11	0.2105 (3)	0.9607 (7)	−0.1694 (4)	0.0253 (11)	
H11	0.1536	0.9921	−0.1625	0.030*	
C12	0.2405 (3)	0.9648 (7)	−0.2726 (4)	0.0272 (11)	
H12	0.2038	0.9993	−0.3347	0.033*	
C13	0.3230 (3)	0.9187 (7)	−0.2832 (3)	0.0233 (10)	
H13	0.3430	0.9218	−0.3526	0.028*	
C14	0.3777 (3)	0.8671 (6)	−0.1917 (4)	0.0214 (10)	
C15	0.5066 (3)	0.7679 (7)	−0.1146 (3)	0.0222 (10)	
H15	0.5631	0.7319	−0.1202	0.027*	

### Atomic displacement parameters (Å^2^)

**Table:**

	*U*^11^	*U*^22^	*U*^33^	*U*^12^	*U*^13^	*U*^23^
Cl1	0.0337 (8)	0.0532 (9)	0.0288 (7)	0.0025 (6)	−0.0040 (5)	0.0098 (6)
Cl2	0.0250 (7)	0.0496 (9)	0.0422 (8)	0.0038 (6)	0.0012 (6)	0.0045 (6)
N1	0.022 (2)	0.029 (2)	0.0190 (19)	0.0013 (17)	0.0001 (16)	−0.0014 (17)
N2	0.025 (2)	0.037 (2)	0.0130 (19)	0.0015 (19)	0.0014 (16)	0.0009 (17)
N3	0.020 (2)	0.026 (2)	0.0155 (18)	−0.0015 (17)	−0.0006 (15)	−0.0013 (16)
N4	0.026 (2)	0.030 (2)	0.0177 (19)	0.0012 (19)	0.0034 (16)	−0.0016 (17)
C1	0.025 (3)	0.024 (2)	0.027 (3)	0.003 (2)	0.000 (2)	0.000 (2)
C2	0.024 (2)	0.032 (3)	0.015 (2)	0.003 (2)	−0.0031 (18)	0.000 (2)
C3	0.033 (3)	0.021 (2)	0.035 (3)	−0.009 (2)	−0.007 (2)	0.002 (2)
C4	0.024 (2)	0.030 (3)	0.029 (3)	−0.002 (2)	0.000 (2)	0.001 (2)
C5	0.031 (3)	0.033 (3)	0.027 (3)	0.000 (2)	0.002 (2)	0.000 (2)
C6	0.028 (3)	0.033 (3)	0.026 (3)	−0.005 (2)	0.002 (2)	−0.003 (2)
C7	0.027 (3)	0.030 (3)	0.013 (2)	0.002 (2)	−0.0005 (18)	−0.0013 (19)
C8	0.027 (2)	0.021 (2)	0.012 (2)	−0.001 (2)	0.0000 (18)	−0.0011 (17)
C9	0.027 (2)	0.022 (2)	0.015 (2)	−0.002 (2)	0.0009 (18)	−0.0037 (18)
C10	0.023 (2)	0.029 (3)	0.015 (2)	−0.001 (2)	0.0025 (18)	−0.0006 (19)
C11	0.023 (2)	0.028 (3)	0.024 (3)	0.001 (2)	0.0002 (19)	−0.002 (2)
C12	0.035 (3)	0.027 (3)	0.017 (2)	−0.002 (2)	−0.009 (2)	0.0006 (19)
C13	0.027 (3)	0.032 (3)	0.011 (2)	0.002 (2)	0.0001 (18)	−0.0005 (19)
C14	0.022 (2)	0.024 (2)	0.019 (2)	0.001 (2)	0.0012 (18)	−0.0025 (19)
C15	0.025 (2)	0.028 (2)	0.014 (2)	0.003 (2)	−0.0018 (17)	−0.0006 (19)

### Geometric parameters (Å, º)

**Table:**

Cl1—C3	1.733 (5)	C5—C6	1.393 (7)
Cl2—C4	1.725 (5)	C5—H5	0.9500
N1—C7	1.277 (6)	C6—H6	0.9500
N1—N2	1.375 (5)	C7—H7	0.9500
N2—C8	1.361 (6)	C8—C15	1.421 (6)
N2—H2N	0.92 (5)	C9—C10	1.402 (6)
N3—C8	1.323 (6)	C9—C14	1.420 (6)
N3—C9	1.375 (6)	C10—C11	1.383 (6)
N4—C15	1.312 (6)	C10—H10	0.9500
N4—C14	1.378 (6)	C11—C12	1.414 (6)
C1—C6	1.392 (7)	C11—H11	0.9500
C1—C2	1.400 (6)	C12—C13	1.381 (7)
C1—C7	1.453 (6)	C12—H12	0.9500
C2—C3	1.406 (7)	C13—C14	1.406 (6)
C2—H2	0.9500	C13—H13	0.9500
C3—C4	1.385 (7)	C15—H15	0.9500
C4—C5	1.401 (7)		
			
C7—N1—N2	116.3 (4)	C1—C7—H7	119.4
C8—N2—N1	120.1 (4)	N3—C8—N2	116.1 (4)
C8—N2—H2N	118 (3)	N3—C8—C15	121.9 (4)
N1—N2—H2N	122 (3)	N2—C8—C15	122.0 (4)
C8—N3—C9	116.6 (4)	N3—C9—C10	119.1 (4)
C15—N4—C14	116.8 (4)	N3—C9—C14	121.3 (4)
C6—C1—C2	119.0 (4)	C10—C9—C14	119.6 (4)
C6—C1—C7	122.6 (4)	C11—C10—C9	120.2 (4)
C2—C1—C7	118.3 (4)	C11—C10—H10	119.9
C1—C2—C3	119.6 (4)	C9—C10—H10	119.9
C1—C2—H2	120.2	C10—C11—C12	120.3 (4)
C3—C2—H2	120.2	C10—C11—H11	119.9
C4—C3—C2	120.8 (4)	C12—C11—H11	119.9
C4—C3—Cl1	120.8 (4)	C13—C12—C11	120.1 (4)
C2—C3—Cl1	118.4 (4)	C13—C12—H12	119.9
C3—C4—C5	119.5 (5)	C11—C12—H12	119.9
C3—C4—Cl2	121.4 (4)	C12—C13—C14	120.3 (4)
C5—C4—Cl2	119.0 (4)	C12—C13—H13	119.9
C6—C5—C4	119.5 (5)	C14—C13—H13	119.9
C6—C5—H5	120.2	N4—C14—C13	120.0 (4)
C4—C5—H5	120.2	N4—C14—C9	120.5 (4)
C1—C6—C5	121.4 (5)	C13—C14—C9	119.5 (4)
C1—C6—H6	119.3	N4—C15—C8	122.9 (4)
C5—C6—H6	119.3	N4—C15—H15	118.5
N1—C7—C1	121.1 (4)	C8—C15—H15	118.5
N1—C7—H7	119.4		
			
C7—N1—N2—C8	−179.9 (4)	N1—N2—C8—C15	2.6 (7)
C6—C1—C2—C3	−1.9 (7)	C8—N3—C9—C10	177.5 (4)
C7—C1—C2—C3	179.0 (4)	C8—N3—C9—C14	−1.5 (6)
C1—C2—C3—C4	−0.1 (7)	N3—C9—C10—C11	−179.8 (4)
C1—C2—C3—Cl1	−179.2 (4)	C14—C9—C10—C11	−0.8 (7)
C2—C3—C4—C5	2.0 (7)	C9—C10—C11—C12	0.2 (7)
Cl1—C3—C4—C5	−178.9 (4)	C10—C11—C12—C13	0.2 (7)
C2—C3—C4—Cl2	−177.1 (4)	C11—C12—C13—C14	0.1 (7)
Cl1—C3—C4—Cl2	1.9 (6)	C15—N4—C14—C13	−179.2 (4)
C3—C4—C5—C6	−1.9 (8)	C15—N4—C14—C9	−0.1 (7)
Cl2—C4—C5—C6	177.2 (4)	C12—C13—C14—N4	178.3 (4)
C2—C1—C6—C5	2.0 (7)	C12—C13—C14—C9	−0.8 (7)
C7—C1—C6—C5	−179.0 (5)	N3—C9—C14—N4	1.0 (7)
C4—C5—C6—C1	−0.1 (8)	C10—C9—C14—N4	−178.0 (4)
N2—N1—C7—C1	−179.3 (4)	N3—C9—C14—C13	−179.8 (4)
C6—C1—C7—N1	0.7 (7)	C10—C9—C14—C13	1.2 (7)
C2—C1—C7—N1	179.8 (4)	C14—N4—C15—C8	−0.2 (7)
C9—N3—C8—N2	−178.7 (4)	N3—C8—C15—N4	−0.3 (7)
C9—N3—C8—C15	1.2 (6)	N2—C8—C15—N4	179.6 (4)
N1—N2—C8—N3	−177.5 (4)		

### Hydrogen-bond geometry (Å, º)

**Table:**

*D*—H···*A*	*D*—H	H···*A*	*D*···*A*	*D*—H···*A*
N2—H2*N*···N4^i^	0.92 (5)	2.10 (5)	3.013 (5)	171 (4)

Symmetry code: (i) *x*, −*y*+3/2, *z*+1/2.
